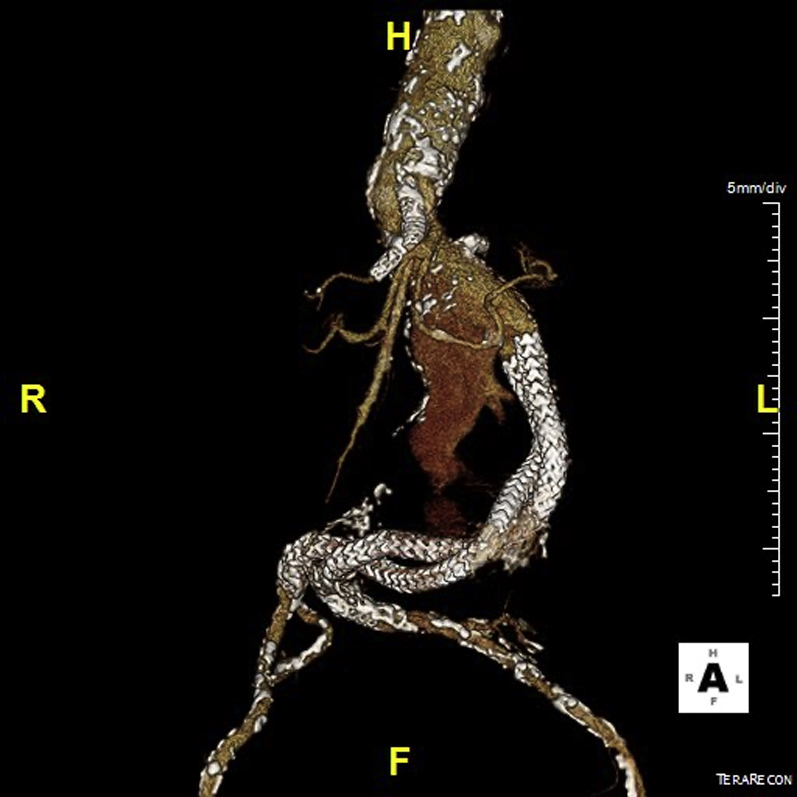# Ongoing migration of chimney endovascular aneurysm sealing

**DOI:** 10.1016/j.jvscit.2020.06.005

**Published:** 2020-06-25

**Authors:** Joshua Field, Shirley Ketting, Geert Willem H. Schurink, Barend M.E. Mees

**Affiliations:** Department of Vascular Surgery, Maastricht University Medical Center, Maastricht, The Netherlands

A 78-year-old woman presented in 2014 with a pulsatile swelling in the abdomen. She had a medical history of chronic obstructive pulmonary disease (Global Initiative for Chronic Obstructive Lung Disease stage 2), reduced renal function (estimated glomerular filtration rate of 59 mL/min/1.73 m^2^), smoking, poor appetite, decreased exercise tolerance, and progressive weight loss over the years with a body mass index of 19 kg/m^2^. On computed tomography angiography (CTA), a 62-mm juxtarenal abdominal aortic aneurysm with associated thrombus in the visceral aorta was identified. She was assessed to be not fit for open repair because of pulmonary status and low body mass index. Because of the size and location of the thrombus in the visceral aorta, the patient was also not anatomically suitable for fenestrated endovascular aneurysm repair. Thus, a chimney endovascular aortic sealing procedure using Nellix (Endologix, Irvine, Calif) prostheses with two renal chimney grafts was performed. The intervention was successful, and postoperative CTA demonstrated adequately positioned and patent stent grafts and chimneys (*A*).

**Figure:**
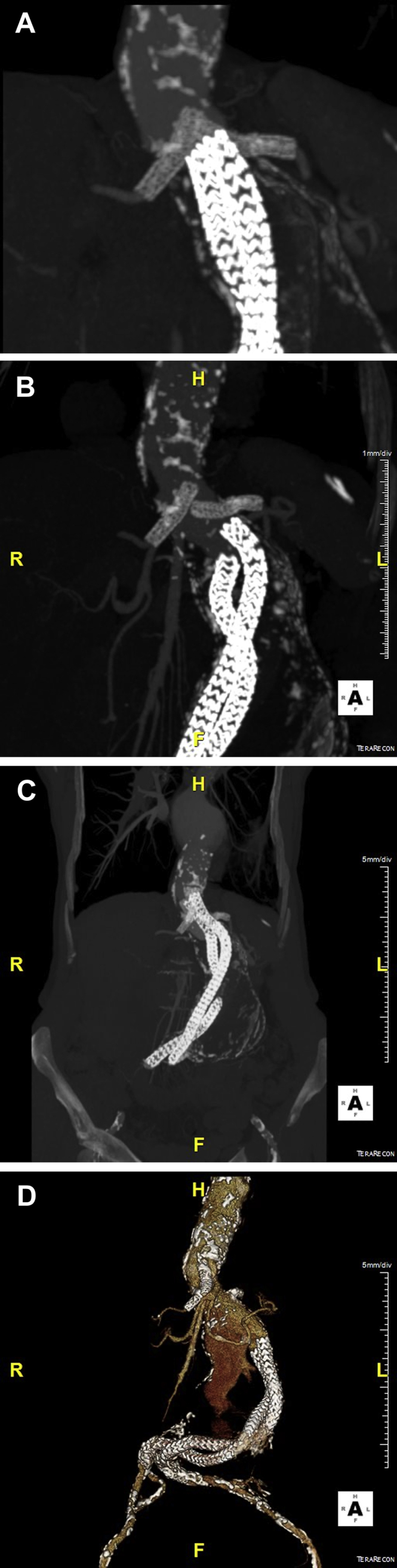

However, in November 2015, regular follow-up CTA (*B*) revealed significant migration of the Nellix stents. Open conversion was not regarded as a feasible option because of the condition of the patient. Therefore, a Nellix-in-Nellix extension including proximal extension of both renal chimney grafts and additional placement of a chimney into the superior mesenteric artery was performed. Again, the intervention was successful, and postoperative CTA demonstrated adequate placement without complications (*C*).

Unfortunately, in 2017, follow-up CTA revealed again migration of the Nellix stents. Because there was no endoleak and all chimney grafts were patent, we employed a watch-and-wait approach. One year later, CTA confirmed continuing migration with additional flattening of the left renal chimney graft, proximal endoleak, and aneurysm growth to 85 mm. Because the patient was asymptomatic and in the same medical condition, we continued a conservative approach. In 2019, 5 years after the initial treatment, she was admitted with severe abdominal pain. CTA confirmed a 100-mm ruptured aneurysm due to ongoing migration of the Nellix stent grafts and showed a 180-degree displaced left renal artery chimney, which surprisingly remained perfused (*D*/Cover). She died with comfort care the next day. The patient’s family consented to publication of this report.

## Figures and Tables

**Cover image:**